# Identification and Validation of a Stromal EMT Related LncRNA Signature as a Potential Marker to Predict Bladder Cancer Outcome

**DOI:** 10.3389/fonc.2021.620674

**Published:** 2021-03-04

**Authors:** YiHeng Du, Bo Wang, Xiang Jiang, Jin Cao, Jiang Yu, Yi Wang, XiZhi Wang, HaiTao Liu

**Affiliations:** ^1^ Department of Urology, Suzhou Kowloon Hospital, Shanghai Jiaotong University School of Medicine, Suzhou, China; ^2^ Department of Pathology, Suzhou Kowloon Hospital, Shanghai Jiaotong University School of Medicine, Suzhou, China; ^3^ Department of Urology, Shanghai General Hospital, Shanghai Jiaotong University School of Medicine, Shanghai, China

**Keywords:** bladder cancer, epithelial to mesenchymal transition, long non-coding RNAs, tumor microenvironment, immunosuppression

## Abstract

Bladder cancer (BLCA) has become one of the most common malignant tumors in the genitourinary system. BLCA is one of the tumors considered suitable for immunotherapy because of the large proportion of immune cells in TME. Epithelial to mesenchymal transition (EMT) is closely related to tumor immunity through its crosstalk with immune cells. A recent study validated that EMT-related genes were mainly expressed by stromal cells and could influence immunotherapy responsiveness. Stromal EMT-related gene signature was also demonstrated to affect the prognosis of multiple tumors, including BLCA. To further explore the prognostic roles of stromal components, we performed a comprehensive analysis of LncRNAs closely associated with stromal EMT-related genes in the TCGA BLCA cohort. We identified a signature including five stromal EMT gene-related LncRNAs that showed significant prognostic value for BLCA patients. By the CIBERSORT and MCP-COUNTER algorithm, we found the signature was markedly correlated with infiltrated immune cells and stromal components of the tumor microenvironment, which may further influence patient’s responsiveness to immune checkpoint blockade therapy. Through immunohistochemical analysis, we confirmed the correlation of the signature with macrophages M2 and CAFs. Meanwhile, key genes related to these LncRNAs, including VIM, MMP2, were also differentially expressed in the stromal components concerning the signature. Our research confirmed the prognostic and immune-associated role of stromal EMT-related LncRNAs. Meantime, we further confirmed that EMT-related genes were mainly expressed in stromal components. Targeting these LncRNAs as well as their related stromal EMT genes may provide potential therapeutic targets for BLCA immunotherapy and precision medicine.

## Introduction

Bladder cancer (BLCA) has become one of the most common malignant tumors in the genitourinary system (1). Transitional cell carcinoma is the most common pathological type of BLCA. According to the invasion depth, BLCA can be divided into non-muscle invasive(NMIBC) and muscle invasive bladder cancer(MIBC).NMIBC can be treated by transurethral resection of bladder tumor (TURBT) and has a favorable prognosis but still faces recurrence risk. MIBC is poorly treated clinically and often with radical surgery supplemented by postoperative chemotherapy and radiotherapy (2). The 5-year survival rate of MIBC patients is only 50% (3). Due to the rapid development of immunotherapy, immune checkpoint inhibitors for advanced BLCA have recently received significant attention. However, the responsiveness to existing immune checkpoint inhibitors is limited among BLCA patients, partly due to a complex heterogeneous tumor microenvironment (TME). Further study of TME is of great importance for BLCA immunotherapy.

TME is a complex and integrated system mainly composed of stromal cells and infiltrated immune cells. Emerging evidence suggests that the stromal components can shape antitumor immunity and affect immunotherapy responsiveness, thus promoting tumors’ malignant development (4). The TME is particularly important in BLCA because of the overwhelming evidence that BLCA represents a growing number of solid tumors characterized by a significant number of stromal and immune cells in the TME (5, 6). The heterogeneity of TME in BLCA is closely related to patients’ different response rates to immunotherapy, in which expression of stromal epithelial to mesenchymal transition (EMT) related genes plays a vital role (7).

EMT has been defined as a dynamical process with intermediate states (8). A complicated regulatory network is engaged in EMT’s dynamic procedure at different levels, which further remodels the tumor extracellular matrix and promotes tumor metastasis. Although EMT is a biological process unique to tumor cells and has been studied extensively *in vitro* and in model organisms, evidence for this phenomenon in human tumors has been limited (9). In contrast, accumulating evidence showed that the up-regulation of EMT-related genes in bulk tumors was driven by expression changes in fibroblasts rather than in epithelial tumor cells (10). For example, Li et al. demonstrated that EMT-related genes were up-regulated only in a subpopulation of cancer-associated fibroblasts (CAFs) through single-cell RNA sequencing (11). A recent study also indicated that stromal EMT-related gene expression might alter T-cell infiltration level, which might eventually impact the responsiveness to immune therapy and patient’s survival (7). These findings illuminated intertwined and complicated crosstalks between the stromal components and the EMT process in the development of cancer immune evasion, making the stromal components promising therapeutic targets for cancer immunotherapy.

LncRNAs are novel, potential therapeutic targets and biomarkers for cancer treatments (12). Moreover, researchers have demonstrated that LncRNAs obtain more specificity on indicating actual tumor condition than other types of markers (13). Previous studies have already discussed the prognostic value of stromal EMT-related genes in BLCA (7, 14), but the roles of stromal EMT-associated LncRNAs (sEMTLncs) have rarely been reported. Therefore, we carried out this present study to explore the function of sEMTLncs and seek their potential application for predicting BLCA outcome. We constructed a stromal EMT-related LncRNA prognostic signature (sEMTLS) in the present study, which showed good predictive accuracy for BLCA outcome. By adopting the CIBERSORT and MCP-COUNTER algorithm, we further found the sEMTLS was significantly associated with the levels of CAFs and tumor infiltrated immune cells (TIICs). Through immunohistochemical validation of the expressions of ACTA2, CD206, and these sEMTLnc-targeted EMT genes, we found that with the different expression levels of these sEMTLncs, these genes were also differentially expressed in the stromal components. These results shed light on the dual regulatory effect of sEMTLncs on both stromal and immune components in BLCA and laterally confirmed the EMT-related genes’ expression was indeed in tumor stromal but not in epithelial tumor cells. Moreover, the ImmuneCell AI database (15) predicted that the expression of these sEMTLncs might also affect BLCA patients’ responsiveness to immune checkpoint blockade (ICB) treatment. Targeting these sEMTLncs as well as their related stromal EMT genes may provide potential therapeutic targets for BLCA immunotherapy and precision medicine.

## Methods and Materials

### Raw Data Acquisition

430 samples of transcriptome profiling data, including 19 normal samples, 411 tumor samples, and the corresponding clinical data of 405 bladder transitional cell papilloma and carcinoma patients were downloaded from the TCGA database (https://portal.gdc.cancer.gov/). Finally, 403 BLCA patients were selected and randomly arranged into training (n = 203) and testing groups (n = 200) for further study. Patients’ baseline information was listed in **Table 1**.

**Table 1:** Clinical characteristic of patients in training, testing and entire groups.

characteristic		Entire group (N = 403)	Training group (N = 203)	Testing group (N = 200)
**age**	≤65	159 (39.5%)	82(40.4%)	77(38.5%)
	>65	244 (60.5%)	121(59.6%)	123(61.5%)
**gender**	male	298 (73.9%)	145(71.4%)	153(76.5%)
	female	105 (26.1%)	58(28.6%)	47(23.5%)
**grade**	low	20 (5.0%)	10(5.0%)	10(5%)
	high	380 (95.0%)	191(95.0%)	189(95%)
**stage**	stages I–II	130 (32.4%)	72(35.8%)	58(29.0%)
	stage III	138 (34.4%)	64(31.8%)	74(37.0%)
	stage IV	133 (33.2%)	65(32.4%)	68(34.0%)
**T**	T1–T2	122 (32.9%)	68(36.6%)	54(29.2%)
	T3–T4	249 (67.1%)	118(63.4%)	131(70.8%)
**N**	N0	234 (64.6%)	118(64.8%)	116(64.4%)
	N1	46 (12.7%)	25(13.7%)	21(11.7%)
	N2	75 (20.7%)	38(20.9%)	37(20.6%)
	N3	7 (2.0%)	1(0.6%)	6(3.3%)
**M**	M0	193(94.6%)	103(94.5%)	90(94.7%)
	M1	11(5.4%)	6(5.5%)	5(5.3%)

### Stromal Scoring and Differential Expressed Genes (DEGs) Screening

R language version 4.0.2 loaded with ESTIMATE package was used to calculate the scores of the immune and stromal component in TME for each sample of BLCA patients. After estimation, stromal score, immune score, and estimate score were obtained, representing the abundance of stromal, immune components, and the total. Then, samples were categorized into high and low-stromal score groups based on all samples’ median stromal score. Package limma was applied to perform differential analysis of the gene expression; DEGs were screened by comparing the gene expressions in samples between high and the low stromal score groups. DEGs with fold change more than 1 after transformation of log2 and FDR <0.01 were considered significant.

### sEMTLnc Acquisition

The Molecular Signatures Database v7.2 (hallmark_epithelial_mesenchymal_transition M5930, http://www.broadinstitute.org/gsea/msigdb/index.jsp) was used to provide 200 EMT-related genes. By Pearson correlation analysis, we defined LncRNAs co-expressed with EMT-associated genes as EMTLnc, with |R| >0.4 and P <0.001. sEMTLncs were further defined as EMTLnc that significantly affected the stromal score, obtained by taking the intersection of stromal DEGs and EMTLnc. Similarly, immune-related LncRNAs (ImmLncs) were obtained through co-expression analysis with genes in immune-related genesets (Immune system process M13664, Immune response M19817, The Molecular Signatures Database v7.2, http://www.broadinstitute.org/gsea/msigdb/index.jsp). ImmLncs were used to check the involvement of sEMTLnc in the immune process.

### sEMTLnc Signatures

sEMTLncs affecting the survival of BLCA were selected by univariate COX analysis using R software survival packages (P < 0.01). Lasso and multivariate cox regression were further used to construct the sEMTLS. Hazard ratio (HR) was used to classify sEMTLnc into the protective (HR < 1) and deleterious (HR > 1) portion (**Table 2**). Patients were classified into high- and low-risk groups based on the medium riskscore of the signature. The riskscore was calculated by the formula as followed:

$$riskscore=\sum_{i=1}^{n}Coef\left(i\right)*\left(expression\;of\;sEMTLnc\left(i\right)\right)$$

*Coef: coefficient of the multi-Cox regression

**Table 2:** EMT-related LncRNAs identified from Cox regression analysis.

Symbol	Description	Multi-Cox regression	Uni-Cox regression			
		coefficient	HR	HR.95L	HR.95H	P-value
AL583785.1		0.056	1.079	1.027	1.133	0.002
TMEM51-AS1	TMEM51 antisense RNA 1	−0.353	0.709	0.555	0.906	0.006
AC073534.1		−0.793	0.400	0.211	0.762	0.005
LINC01711	long intergenic non-protein coding RNA 1711	0.062	1.101	1.046	1.159	<0.001
LINC02446	long intergenic non-protein coding RNA 2446	−0.567	0.684	0.515	0.909	0.009

### Survival Analysis

R language v4.0.2 with package survival and survminer were used for survival analysis. Kaplan–Meier survival analysis was used for analyzing the survival difference between different groups. P-value of the log-rank test less than 0.05 were considered significant.

### Time and Multi-ROC Curves

ROC curves analyzed the predictive accuracy of the signature on BLCA overall survival at 1, 3 and 5 years. Package timeROC of R language v4.0.2 was used for plotting timeROC curves. Independent risk analysis of different clinical characteristics, including age, gender, stage, T classification, and risk score on predicting 1-year overall survival, was conducted by Package survivalROC (**Table 3**).

**Table 3:** Independent analysis by univariate and multivariate Cox regression of training, testing, and entire groups.

Uni-Cox regression					multi-Cox regression				
**Training group**									
Variables	HR	HR.95L	HR.95H	P-value	Variables	HR	HR.95L	HR.95H	P-value
Age	2.344	1.358	4.045	0.002	Age	2.266	1.293	3.972	0.004
Gender	0.863	0.530	1.406	0.555	Gender	0.935	0.564	1.549	0.794
Stage	1.953	1.441	2.645	<0.001	Stage	1.623	1.130	2.332	0.009
T	1.911	1.363	2.678	<0.001	T	1.495	0.973	2.298	0.067
RiskScore	1.141	1.093	1.191	<0.001	RiskScore	1.124	1.075	1.175	<0.001
**Testing group**									
Variables	HR	HR.95L	HR.95H	P-value	Variables	HR	HR.95L	HR.95H	P-value
Age	1.649	0.984	2.763	0.058	Age	1.791	1.042	3.077	0.035
Gender	0.789	0.457	1.361	0.394	Gender	0.624	0.354	1.099	0.103
Stage	1.936	1.371	2.733	<0.001	Stage	1.775	1.192	2.644	0.005
T	1.710	1.183	2.471	0.004	T	1.210	0.783	1.871	0.391
RiskScore	1.397	1.202	1.624	<0.001	RiskScore	1.325	1.127	1.558	0.001
**Entire group**									
Variables	HR	HR.95L	HR.95H	P-value	Variables	HR	HR.95L	HR.95H	P-value
Age	1.960	1.348	2.850	<0.001	Age	1.919	1.315	2.799	0.001
Gender	0.840	0.586	1.204	0.343	Gender	0.780	0.540	1.125	0.183
Stage	1.943	1.546	2.442	<0.001	Stage	1.745	1.338	2.277	<0.001
T	1.774	1.387	2.269	<0.001	T	1.292	0.957	1.744	0.094
RiskScore	1.157	1.115	1.200	<0.001	RiskScore	1.137	1.094	1.182	<0.001

### Principal Component Analysis (PCA) and Nomogram Construction

PCA was used to cluster the samples based on the expression of sEMTLnc. A 3D scatterplot visualized patients’ distribution. Nomogram was constructed by including the expression level of the sEMTLnc in the signature. A calibration plot was used to explore the calibration and discrimination of the nomogram.

### GO, KEGG, and GSEA Enrichment Analysis

DEGs between the high- and low-risk groups of sEMTLS were used for GO and KEGG enrichment analysis. The Hallmark_Epithelial_Mesenchymal_Transition gene set was used for GESA between the low-risk and high-risk group, which was performed using the GSEA software (version 4.1.0) obtained from the Broad Institute. NOM p <0.05 and False Discovery Rate (FDR) q <0.05 were considered to be significant.

### Calculation of TIIC Levels and CAFs Abundance

The CIBERSORT algorithm was used for calculating the levels of 22 different types of TIICs in all tumor samples; Samples with a p-value less than 0.05 were selected for the following analysis. The MCP-COUNTER algorithm (16, 17), provided by TIMER 2.0 (http://timer.cistrome.org/) (18), was applied for calculating the abundance of TME components, including endothelial cells and CAFs.

### ICB Treatment Reactiveness Predicting

Immune cell abundance identifier (Immune cell AI, http://bioinfo.life.hust.edu.cn/ImmuCellAI) was applied to estimate the difference of immune cell infiltration in low- and high-risk groups. Patients’ response to ICB therapy was then predicted based on the estimation of immune cell infiltration levels.

### Real-Time Quantitative PCR

According to the manufacturer’s instructions, we used triazole (Invitrogen) to extract total RNA from all recruited BLCA samples. cDNA Synthesis Kit (Osaka, Japan of TaKaRa) combining with RNA (1 μg) was utilized to reverse-transcribed cDNA. The quantitative polymerase chain reaction (qPCR), using the SYBR-Green method (TaKaRa), was performed on an ABI 7500 real-time PCR system (Applied Biosystems). The relative expression level of each lncRNA was calculated by the 2^−ΔΔCt^ method after normalizing to *β*-actin level. The forward and reverse primer sequences are shown in **Table 4**.

**Table 4:** The primer sequences of sEMTLnc.

AC073534.1	Forward(5′–3′)	TCACCTCAGCCAGCAGAAAC
	Reverse(5′–3′)	GGTGTTGACCATCTGTGGACT
TMEM51-AS1	Forward(5′–3′)	CAACAAGACCGAGCCAGGAG
	Reverse(5′–3′)	GCCCCGTCAGTGACTCATAG
AL583785.1	Forward(5′–3′)	GTGGTGCTTTTGCCTACTTGG
	Reverse(5′–3′)	TGGGCATACATCTTGAAGGGT
LINC02446	Forward(5′–3′)	AGCGGAGTGCAAAATGAAGTG
	Reverse(5′–3′)	CAATCCCACACAGGGTGTCC
LINC01711	Forward(5′–3′)	CTGGTCTGGAGCCGTTTCTC
	Reverse(5′–3′)	ATCCATCCTTGACCCTCGGA
*β*-actin	Forward(5′–3′)	AAACGTGCTGCTGACCGAG
	Reverse(5′–3′)	TAGCACAGCCTGGATAGCAAC

### Immunohistochemistry Analysis

The gene expression in tumor tissues was detected using the BenchMark GX automatic multifunctional immunohistochemical staining system (Roche, Switzerland) with OptiView DAB Detection Kit (Ventana, USA) according to the manufacturer’s instructions. The staining’s straightforward procedures were listed as follows: deparaffinization and epitope retrieval in cell conditioner for 90 min. Short (8 min), mild (30 min), and standard (60 min) cell conditioning was performed after epitope retrieval. Primary antibodies were then incubated with the section for 32 min followed by biotinylated anti-IgG antibody and streptavidin–biotinylated-complex horseradish peroxidase. Hematoxylin was used for counterstaining and Bluing Reagent for post counterstaining. Details of the analyzed genes were listed in **Table 5**.

**Table 5:** Genes used in immunohistochemical analysis.

Primary antibody	Description	Role of gene	Manufacturer	Catalog	Dilution
ACTA2	Actin Alpha 2, smooth muscle	a marker of CAFs	Abcam	ab7817	1:100
CD206	Mannose receptor C-type 1	a marker of Macrophages M2	Abcam	ab252921	1:4000
MMP-2	Matrix metallopeptidase 2	Key sEMTLnc-related gene	Abcam	ab97779	1:200
VIM	Vimentin	Key sEMTLnc-related gene	Abcam	ab92547	1:200
CALU	Calumenin	Key sEMTLnc-related gene	Abcam	ab137019	1:250

### Statistics Analysis

Correlation of sEMTLS with infiltrated immune cells was analyzed with Pearson correlation test; p <0.01 was considered significant. Kaplan–Meier curve with log-rank test was used to evaluate the OS between different groups. The Wilcoxon test examined the differences for variables of two groups. Kruskal test estimated statistical significance for variables of more than two groups. Univariate and multivariate Cox regression analyses were displayed to verify the independent prognostic factors for BLCA. Fisher exact test was used to calculate the difference of ICB response between high- and low-risk groups. Two-sided P-value <0.05 was considered significant. R language v4.0.2 was used for all statistical analyses.

## Results

### 72 sEMTLnc Were Identified for Signature Construction

**Figure 1:**
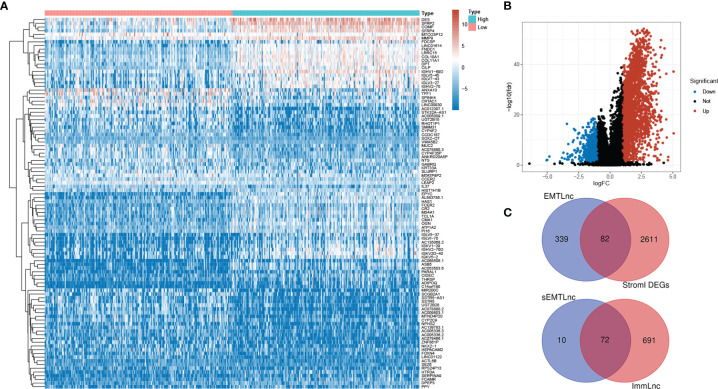
Screening for sEMTLnc. **(A)** The top 50 of both up- and down-regulated stromal DEGs with the most significant differences were represented by the heatmap. **(B)** The distribution of all DEGs on the two dimensions of -log (FDR) and logFC was depicted in the volcano plot. **(C)** The intersection of stromal DEGs and EMTLnc identified 82 sEMTLncs, 72 out of 82 sEMTLncs were involved in the immune process.

After we scored the stromal components by the ESTIMATE algorithm, DEGs between low and high stromal score groups were screened. 2,693 DEGs were identified. The top 50 of both up- and down-regulated genes with the most significant fold changes were represented by the heatmap (**Figure 1A**). The distribution of all DEGs on the two dimensions of -log10(FDR) and logFC was depicted in the volcano plot (**Figure 1B**). 421 EMTLncs were enrolled by co-expression with EMT-related genes. 82 sEMTLncs were selected after the intersection of the EMTLnc with the DEGs (**Figure 1C**). After the intersection with ImmLnc, 72 out of 82 sEMTLncs were confirmed to be highly co-expressed with genes related to the immune process, which further demonstrated the close relationships between EMT and the immune process. These 72 sEMTLncs were chosen for the construction of sEMTLnc signature.

### sEMTLS Efficiently Predicted the Clinical Outcome of BLCA Patients in the TCGA Cohort

**Figure 2:**
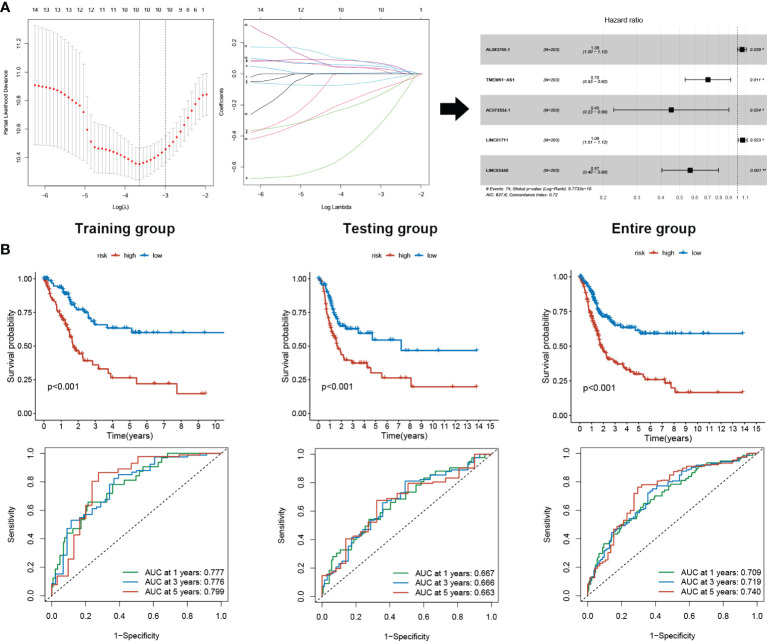
sEMTLS construction and prognostic value validation. **(A)** sEMTLS were constructed by uni-cox regression, lasso regression, and multi-cox regression, which included three protective and two hazardous sEMTLnc. **(B)** Kaplan–Meier survival analysis suggested a lower OS in high-risk groups. ROC curve showed good accuracy of the sEMTLS in predicting 1, 3 and 5 years patients’ OS.

403 TCGA BLCA patients were randomly assigned to the training (203 patients) and the testing (200 patients) groups (**Table 1**). Five sEMTLncs were included in the signature, namely AL583785.1, TMEM51-AS1, AC073534.1, LINC01711, and LINC02446 (**Table 2**). A forest plot illustrated the corresponding HRs and 95% CIs for each sEMTLnc (**Figure 2A**). Patients were classified into a high-risk group and a low-risk group based on the training group’s median risk score. Patients’ overall survival (OS) in the high-risk group was significantly shorter than that in the low-risk group (p < 0.001). Time ROC curve showed good predictive accuracy with AUC of 0.777, 0.776, and 0.799 for predicting 1, 3 and 5 years’ OS. Subsequently, we validated the sEMTLS in testing and the entire groups. Statistically, significant OS differences were observed between the high- and low-risk groups in the testing (p < 0.001) and the entire group (p < 0.001). AUC of time ROC curves in the testing and the entire group were 0.667, 0.666, 0.663, and 0.709, 0.719, 0.740 for predicting 1, 3, and 5 years’ OS, respectively (**Figure 2B**).

### The Risk Score of sEMTLS Could Essentially Predict the Clinical Status of BLCA Patients

**Figure 3:**
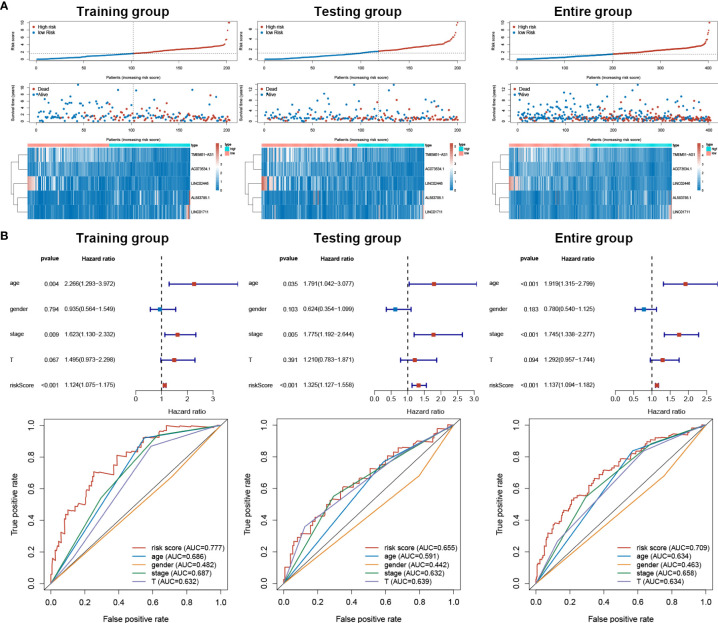
sEMTLS correlated with mortality risk and could be used as an independent prognostic factor for BLCA. **(A)** Patients’ mortality status and sEMTlnc expression in each patient were plotted according to the ordered risk score of sEMTLS. **(B)** Multivariate and ROC curves confirmed sEMTLS as an independent prognostic factor for BLCA.

**Figure 4:**
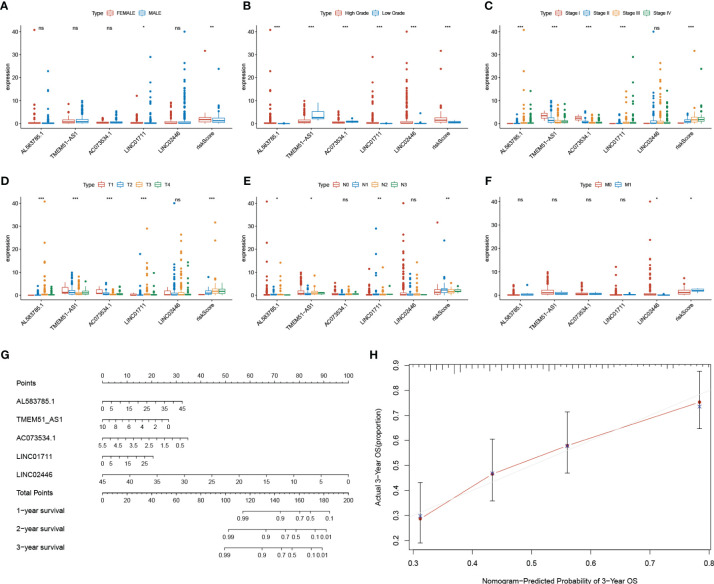
Relationships between sEMTLS and clinical features of BLCA patients and nomogram of sEMTLS. **(A–F)** sEMTLS was significantly related to clinical features, including gender, grade, staging and TNM classification. **(G, H)** Nomogram of sEMTLS showed high consistency with the actual 3-year survival.

Based on our signature, the mortality risk of patients in each group climbed with the risk score increased. The expression levels of AL583785.1 and LINC01711 were elevated, while TMEM51-AS1, AC073534.1, and LINC02446 expressed decreasingly as risk score increased (**Figure 3A**). Combined with other clinical and demographic characteristics of BLCA patients, the risk score was identified by multivariate cox regression analysis to be an independent prognostic factor for BLCA patients, with a hazard ratio of 1.124(1.075–1.175), 1.325(1.127–1.558), 1.137(1.094–1.182) in training, testing and entire group respectively (**Figure 3B**). The ROC curve validated the prognostic accuracy of the risk score. The AUC of the risk score was higher than any other clinical and demographic characteristics in each group, which further suggested the risk score could be an independent prognostic factor (**Table 3**). To further validate the prognostic value and explore the broad applicability of sEMTLS, we analyzed the relationships between sEMTLS with different clinical features, including patient age, tumor grade, staging, and TNM classification in the entire group. The risk score was found significantly related to all of these clinical features confirming the significant association of sEMTLS with the progression of BLCA (**Figures 4A–F**). Last, we constructed a nomogram of sEMTLS to predict patient survival (**Figure 4G**). The calibration curve indicated that sEMTLS had a high consistency with the actual 3-year survival (**Figure 4H**).

### PCA and Functional Analysis Between High- and Low-Risk Groups of sEMTLS

**Figure 5:**
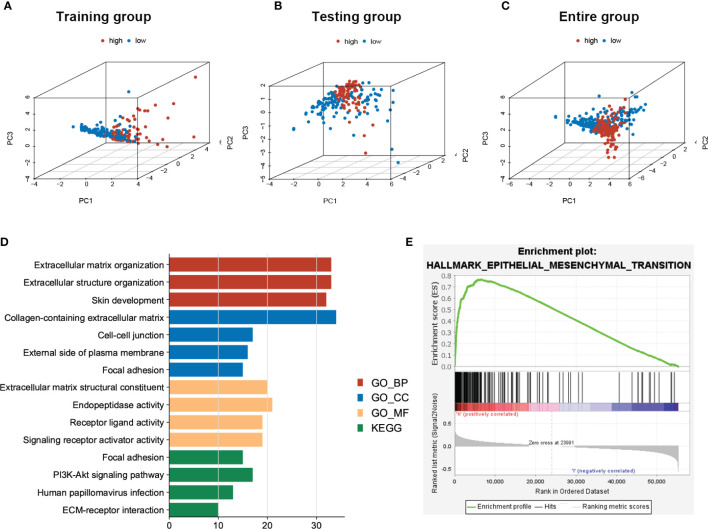
PCA of patients’ distribution and functional analysis between low- and high-risk patients in the entire group. **(A–C)** PCA showed a scattered distribution of patients in testing and the entire groups, while a small portion of overlap was observed in the testing group. **(D)** DEGs between high and low-risk groups majorly enriched in EMT related functions and pathways. **(E)** GSEA confirmed a high EMT activity in high-risk groups.

Based on the expressions of sEMTLnc recruited in the sEMTLS, we employed the principal component analysis (PCA) to provide an overview of different distribution patterns between the low-risk group and the high-risk group. Results indicated a relatively scattered distribution of patients in testing and the entire groups, but with a small overlap in the training group (**Figures 5A–C**). GO and KEGG analysis of DEGs between high and low-risk groups of the entire group were enriched in terms related to extracellular matrix remodeling, including extracellular matrix organization, extracellular matrix structural constituent, and ECM-receptor interaction (**Figure 5D**). GSEA further proved the functional annotation, with the more EMT-related activity in the high-risk group (**Figure 5E**).

### Close Relationships Were Found Between sEMTLS and TIICs

**Figure 6:**
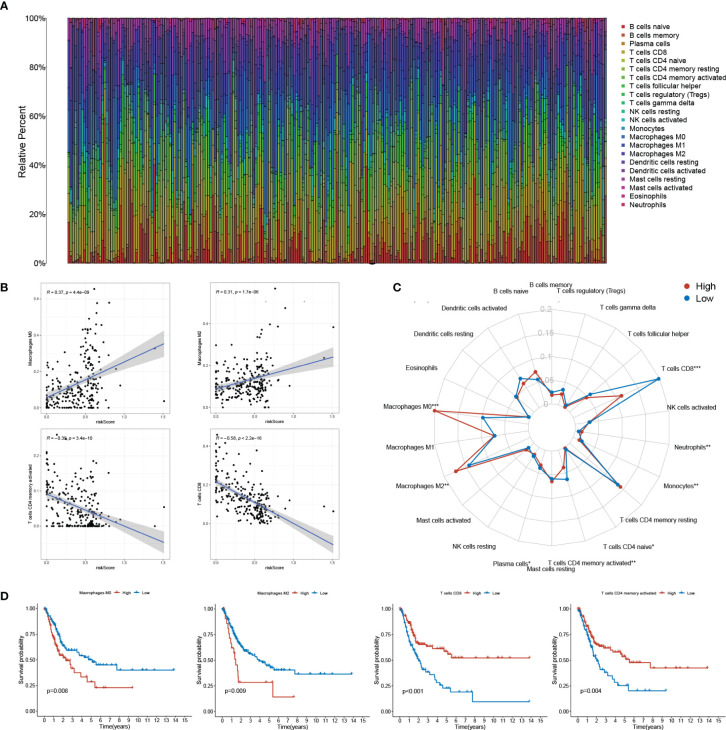
sEMTLS is correlated with TIICs levels, including macrophages and CD8+ T cells. **(A)** The percentage of 22 TIICs in each patient of the entire group was shown in the barplot. **(B, C)** sEMTLS positively related to Macrophages M0 and M2, while negative relationships were observed between sEMTLS and CD8+ T cells and T cell CD4 memory activated. **(D)** All of the four sEMTLS related TIICs affected the OS of BLCA patients. Macrophages M0 and M2 served as detrimental factors, while T cell CD8+ and T cell CD4 memory as protective factors.

Utilizing the CIBERSORT algorithm, we obtained an estimation of the abundances of 22 TIICs. The infiltration proportion of the immune cells in each sample was shown in the barplot (**Figure 6A**). Among all the TIICs, Macrophages M0 and M2 were positively correlated to the risk score while T cell CD8+ and T cell CD4 memory activated exhibited negative correlations (**Figure 6B**). Further, elevated levels of Macrophages M0, M2, and decreased levels of T cell CD8+ and T cell CD4 memory activated were found in the high-risk group when compared with the low-risk group (**Figure 6C**). Combined with the survival time and survival state, all these four TIICs showed significant relations with the OS of BLCA patients, with Macrophages M0, M2 being detrimental factors and T cell CD8+, T cell CD4 memory activated being protective factors (**Figure 6D**). The above results highlighted the association between sEMTLS and TIICs, indicating the immune-modulating role of the sEMTLnc.

### Correlation of sEMTLS With CAFs and Its Predicting Value to the Responsiveness of ICB Treatment

**Figure 7:**
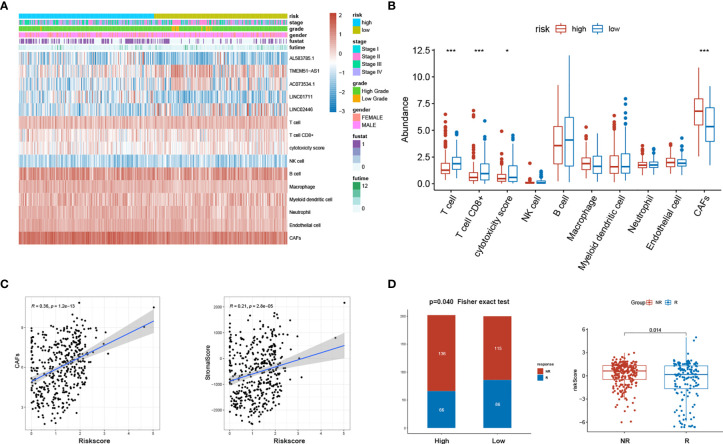
sEMTLS was related to the stromal components and ICB responsiveness. **(A)** Expression of sEMTLnc and TME cells calculated by MCP-COUNTER in each patient was shown in the heatmap. **(B)** Significant lower CD8+ T cells and higher CAFs abundance were observed in the high-risk group. **(C)** The risk score of sEMTLS was positively related to CAFs abundance and the stromal score. **(D)** Patients in the high-risk group earned a lower responsiveness rate to ICB therapy. Risk scores are lower in patients who respond to ICB therapy than those who do not respond.

MCP-COUNTER algorithms calculated CAFs abundance in patients of the TCGA BLCA cohort. The relative abundance of CAFs was represented in the heatmap (**Figure 7A**). After comparing the TME components calculated by MCP-COUNTER between high- and low-risk groups, T cells CD8+ were further confirmed to be reduced in the high-risk group, while CAFs abundance was significantly higher in the high-risk group than in the low-risk group (**Figure 7B**). The risk score was further validated positively correlated with CAFs abundance and the stromal score (**Figure 7C**). Using the immune cell AI database, We found significantly lower ICB treatment responsiveness in high-risk groups than the low-risk group (p=0.040). The risk scores between responders and non-responders also differed with relative higher risk scores in non-responders (p = 0.014) (**Figure 7D**).

### Validation of the Association Between sEMTLS and TME Components in a Clinical Cohort

**Figure 8:**
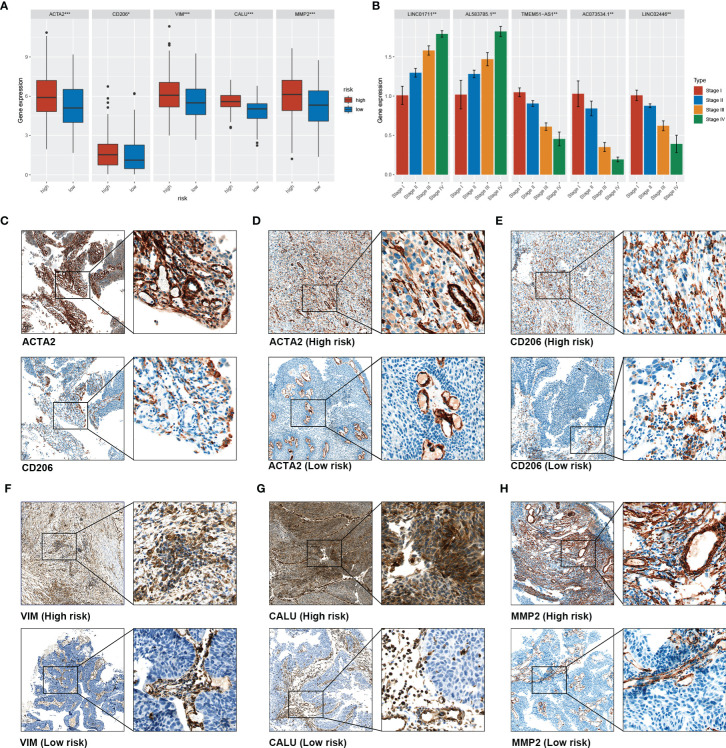
Validation of the association between sEMTLS and TME Components in our clinical cohort. **(A)** the expression level of ACTA2, CD206, VIM, MMP2, and CALU in the high- and low-risk group of the TCGA cohort. **(B)** PCR analysis confirmed that sEMTLncs were differentially expressed between different stages in our clinical validation cohort **(C)**. Co-expression of CD206 and ACTA2 was found in high-risk patients, confirming the association between macrophages M2 and CAFs **(D)** to **(H)**. The expression levels of ACTA2, CD206, VIM, CALU and MMP2 in the high-risk patients were significantly higher than those in the low-risk patients.

A clinical BLCA cohort of 16 patients with different stages was established to validate the correlation between sEMTLS, sEMTLnc targeted key genes, TIICs and CAFs. In our study, a total of five molecules were used for further research, of which CD206 and ACTA2 were used to represent the relative expression of macrophage M2 and CAFs, while three key lncRNA-targeted genes, VIM, CALU and MMP2, were used to detect expression in patients at different risks. The above five molecules in the TCGA cohort were significantly differentially expressed between the high- and low-risk groups (**Figure 8A**). Relative expressions of the five sEMTLnc were analyzed by real-time quantitative PCR. The risk score of each patient was calculated according to the formula. A close relation of sEMTLnc with the clinical stage was found and shown in the barplot (**Figure 8B**). Expression of CD206 was found in areas where ACTA2 was expressed in high risk patients (**Figure 8C**). We further compared the expression of CD206, ACTA2, VIM, CALU and MMP2 between patients with low and high risk scores (**Figures 8D–H**). The results confirmed an elevated expression of all the five genes in high risk group patients. Furthermore, the expressions of ACTA2, VIM, and MMP2, which are markers of CAFs, were mainly found in the stromal area. These results demonstrated the relationship between CAFs and macrophages M2 and highlighted the association of sEMTLS with CAFs and macrophages M2 in BLCA patients. Our immunohistochemical results also revealed that in addition to CAFs, vascular smooth muscle cells also expressed EMT-related genes, which confirmed and complemented the previous findings of Li et al.

## Discussion

BLCA is one of the tumors considered suitable for immunotherapy because of the large proportion of immune cells in TME. Recent advances in treatment indeed demonstrated immune checkpoint blockers on T cells result in significantly improved survival in BLCA (19). However, challenges remain since there are still many BLCA patients who showed low responsiveness to ICB therapy. Over the past decade, EMT has been considered as a pivotal regulator in metastatic progression (20) and therapy resistance (21), including chemo- (22), radio- (23), and targeted therapy resistance. Under the current understanding, the EMT process is profound for regulating immune cells in TME, including CD8+ T cells and macrophages M2 (24). Evidence is now accumulating that such crosstalks might be a critical mechanism in promoting cancer immune escape (25). Recent studies have identified EMT as a dynamic process with an intermediate status, making the EMT process a promising target for therapeutic intervention (26), which may further promote immunotherapy efficacy in low responsive cancer patients (27).

The TME has been characterized as inflammatory and immunosuppressive (28, 29). It owned a variety types of immune and stromal cells. Among the immune cells, macrophages are highly plastic and crucial to the EMT process (30). Previous studies have confirmed that M2 macrophages secrete a series of cytokines that promote EMT and cancer progression *via* multiple signaling pathways, including ZEB1 (31), SNAL1 (32), VIM (33), and TWIST1 (34). Stromal cells dominated by CAFs also participate in the EMT process. One previous study demonstrated that CAFs could increase the expressions of EMT-related genes and differentiated the recruited monocytes into M2 macrophages, further exerting their immunosuppressive roles targeting CD8+ T cells (35). These results are consistent with our finding in this study that macrophage M2 and CAFs are co-expressed in the stromal compartment. Given all the above evidence, we clearly noticed the association between TME components and the EMT process.

Although EMT is a biological process unique to tumor cells has been studied extensively *in vitro* and in model organisms, evidence for this phenomenon in human tumors has been limited (11). A recent study indicated that stromal components of BLCA comprise a key source of EMT-related gene expression. Expression of these stromal genes correlated with T-cell infiltration and impacted response to immune checkpoint blockade, further influencing BLCA patients’ survival (7). In the present study, we found that the expression of sEMTLnc may also affect the infiltration of T-cells and macrophages M2. Immunohistochemical results of these lncRNA-targeted EMT genes also validated that EMT genes were mainly expressed in stromal components. Whereas, questions may raise that since EMT is a unique process of the tumor cells, why EMT related genes were mainly expressed in stromal components but not in tumor cells themselves. Available evidence suggests that the EMT process may not only act in the epithelial tumor cells but also involve intertwined and complex crosstalks between stromal components and tumor cells. Based on the fact that EMT-related genes are only expressed in a subpopulation of CAFs, CAFs have been suggested to even arise from tumor cells undergoing EMT (7). It is difficult to explain these mechanisms by bulk RNA sequencing. Further research is still needed to validate the exact mechanisms between stromal EMT-related gene expression and tumor cells’ EMT process. Perhaps applying single-cell sequencing to explore these EMT-associated genes’ cellular origin could be useful in explaining this intricate process.

On the other hand, LncRNAs are novel, potential therapeutic targets and biomarkers for cancer treatments (12). Moreover, researchers have demonstrated that LncRNAs obtain more specificity on indicating actual tumor condition than other types of markers (13). Previous research indicated that stromal EMT-related genes could provide a predictive role in tumor patients’ prognosis and affect response to ICB therapy in BLCA patients. Could sEMTLnc play a similar role? From the present results, we confirmed the prognostic value of sEMTLnc in BLCA patients. The sEMTLS could be an independent risk factor to patients’ OS. Close correlations between sEMTLS, macrophage M2 and CAFs were also found, suggesting that the combined expression of sEMTLnc was associated with the abundance of macrophages and CAFs. Also, there was a negative correlation between sEMTLS and CD8+ T cells and a significant difference in ICB treatment responsiveness between high and low-risk groups. These results suggested that sEMTLnc, like stromal EMT-related genes, had a significant influence on the immunotherapeutic response and may ultimately affect the prognosis of BLCA.

Using a clinical validation cohort, we further confirmed the relationship between CAFs, macrophage M2 and the sEMTLnc. The key genes related to the sEMTlnc, including VIM, MMP2, and CALU, also expressed differently concerning the risk score. VIM and MMP2 are also markers of CAFs, which promote cancer progression and metastasis (36, 37). Simultaneously, CALU was demonstrated to express significantly higher levels in the metastatic cancer tissues (38). Through our immunohistochemical results, we could see that markers such as ACTA2, VIM, and MMP2 were mostly differentially expressed in the stromal area, including CAFs and vascular smooth muscle cells, but not in the tumor cells between the different risk score groups. These results are consistent with the previous literature (7, 11), while the detection of expression of key genes targeted by these sEMTlnc in vascular smooth muscle cells also suggested that these EMTlnc may be involved in the formation of tumor neovascularization (39). Taken together, we suggest that these sEMTlnc play a key role in TME remodeling and regulation of TIICs, including T cells and macrophages. The expression of sEMTlnc may ultimately influence immune responsiveness and overall survival of BLCA patients. Further studies on sEMTLnc may provide potential therapeutic targets for BLCA immunotherapy and precision medicine.

However, limitations still existed since our results were mainly based on the bioinformatics analysis. Although highly correlated with EMT-related genes, critical questions still existed: Do sEMTLnc expression indeed reflect the biological process of EMT, How do sEMTLnc expression alter T-cells and macrophages M2 infiltration, why sEMTLnc can impact outcomes and affect the responsiveness to ICB treatment in BLCA patients. Simultaneously, It is not sufficient to study sEMTLnc-associated TIICs only by bulk RNA sequencing data based on bioinformatics algorithms, which may lead to different results between different algorithms. The exact association between sEMTLnc, CAFs, macrophages, and T cells still needs to be verified by a series of *in vitro* and *in vivo* experiments, including single-cell RNA sequencing. Besides, due to the limited number of cases in the clinical cohort we included, we still need a larger external clinical cohort to validate the relative expressions of the sEMTLnc and the predictive value of the signature.

## Conclusion

In the present study, we constructed a prognostic signature containing five sEMTLnc, which predicted BLCA patients’ prognosis. Further study on the signature confirmed its significant correlation with the abundance of CAFs, macrophages M2 and CD8+ T cells. Similar to the literature reporting that stromal EMT gene expression affects BLCA prognosis and immunotherapy responsiveness, the combined expression of stromal EMT-related lncRNAs may also affect BLCA outcome and immunotherapy responsiveness. Through immunohistochemical analysis, we laterally verified that EMT-related genes are mainly expressed in the stromal components, including CAFs and vascular smooth muscle cells. The crosstalk between tumor stroma and tumor cell’s EMT process is intricate and requires in-depth study. Further study of stromal EMT-related lncRNAs and their targeted genes will help provide possible new targets for BLCA precision therapy and immunotherapy.

## Data Availability statement

The datasets presented in this study can be found in online repositories. The names of the repository/repositories and accession number(s) can be found in the article/supplementary material.

## Ethics Statement

The studies involving human participants were reviewed and approved by Medical Ethics Committee of Shanghai General Hospital. The patients/participants provided their written informed consent to participate in this study.

## Author Contributions

YD and BW had an equal contribution to this manuscript. HL designed the whole study. YD and BW participated in the bioinformatics and statistical analysis, XJ and JC did the immunohistochemistry analysis. YD performed real-time PCR analysis. YW and JY made the manuscript and figure editing. XW and HL revised the manuscript. All authors contributed to the article and approved the submitted version.

## Funding

This study was funded by the National Natural Science Foundation of China (Grant number: 81972371) and Basic Research on medical and health Application of Suzhou Municipal Science and Technology Bureau (Grant number: SYSD2020076).

## Conflict of Interest

The authors declare that the research was conducted in the absence of any commercial or financial relationships that could be construed as a potential conflict of interest.
